# Light Intensity Dependence of CO_2_ Assimilation Is More Related to Biochemical Capacity Rather than Diffusional Conductance

**DOI:** 10.3390/plants14070986

**Published:** 2025-03-21

**Authors:** Xiaoqian Wang, Qi Shi, Ningyu Liu, Jianxin Cao, Wei Huang

**Affiliations:** 1 Kunming Institute of Botany, Chinese Academy of Sciences, Kunming 650201, China; wangxiaoqian@mail.kib.ac.cn (X.W.); shiqide0720@163.com (Q.S.); liuningyu@mail.kib.ac.cn (N.L.); 2 Yunnan Academy of Forestry and Grassland, Kunming 650201, China

**Keywords:** photosynthesis, light response, Rubisco carboxylation, stomatal conductance, mesophyll conductance

## Abstract

The response of CO_2_ assimilation rate (*A*_N_) to incident light intensity reflects the efficiency of light utilization. The light intensity dependence of *A*_N_ varies widely among different plant species, yet the underlying mechanisms remain poorly understood. To elucidate this issue, we measured the light intensity dependence of gas exchange and chlorophyll fluorescence in twelve tree species. The results indicated that (1) with increasing light intensity, the variation in *A*_N_ was closely related to stomatal conductance (*g*_s_), mesophyll conductance (*g*_m_), the maximum velocity of Rubisco carboxylation (*V*_cmax_), and electron transport rate (ETR); (2) compared with *A*_N_ at sub-saturating light, the increase in *A*_N_ at saturating light was more strongly associated with *V*_cmax_ and ETR than with *g*_s_ and *g*_m_; and (3) the increase in *V*_cmax_ and *A*_N_ from 600 to 2000 μmol photons m^−2^ s^−1^ were positively correlated with the maximum capacity of *V*_cmax_. These findings suggest that *V*_cmax_ is an energy-dependent process that significantly regulates the light intensity dependence of *A*_N_ in plants. This provides valuable insights for crop improvement through the manipulation of *V*_cmax_.

## 1. Introduction

Plants utilize photosynthesis to convert light energy into chemical energy in the form of ATP and NADPH, which are used to assimilate CO_2_ and synthesize organic matter. The maximum *A*_N_ largely varies among different plants due to changes in stomatal conductance (*g*_s_), mesophyll conductance (*g*_m_), and biochemical capacity [1,2,3,4,5]. Under field environments, natural light intensity usually fluctuates during the daytime due to changes in solar position and cloud cover [6,7]. Light intensity significantly influences the CO_2_ assimilation rate (*A*_N_) through effects on *g*_s_, *g*_m_, Rubisco activation state, and electron transport rate (ETR) [8,9,10]. The response of *A*_N_ to incident light intensity plays a significant role in determining plant growth and crop yield [11].

With the increase in light intensity, *A*_N_ usually increases gradually to reach the maximum value at saturating light. Many previous studies have measured the light response curves of *A*_N_ in plants [8,12,13], pointing out that the light saturation point (LSP) of *A*_N_ largely differs between species. For example, LSP is lower than 300 μmol photons m^−2^ s^−1^ in some low-photosynthesis plants, such as *Paphiopedilum* species and *Pleione aurita* [12,13]. By comparison, LSP is approximately 1500 μmol photons m^−2^ s^−1^ in some high-photosynthesis plants, such as tobacco and tomato [10,14]. Although such species-dependent LSP is related to the maximum *A*_N_ under saturating light, mechanisms underlying the variation in light intensity dependence of *A*_N_ have not been well clarified.

At a given incident light intensity, the value of *A*_N_ can be limited by diffusional CO_2_ conductance and/or biochemical capacities [15]. Specifically, *A*_N_ under low light is mainly limited by the availability of ATP and NADPH produced through ETR [16]. With the increase in light intensity, the major limitation of *A*_N_ gradually shifts from ETR to RuBP carboxylation/regeneration [17]. According to the photosynthesis model, ETR = PPFD × Φ_PSII_ × *s*, where PPFD is the photosynthetic photon flux density, Φ_PSII_ is the effective quantum yield of photosystem II (PSII) photochemistry, and *s* is the proportion of light energy absorbed by leaves allocated to PSII, with a typical value of 0.45. At a moderate illumination of 600 μmol photons m^−2^ s^−1^, ETR can theoretically reach 189 μmol photons m^−2^ s^−1^ when Φ_PSII_ is assumed to be 0.7. The value of *A*_N_ can theoretically reach 23.6 μmol CO_2_ m^−2^ s^−1^ based on the assumption that *A*_N_ = ETR/8. Since the saturating *A*_N_ of most species is lower than 23.6 μmol CO_2_ m^−2^ s^−1^, their LSP of *A*_N_ is theoretically lower than 600 μmol photons m^−2^ s^−1^. However, the actual LSP is usually higher than 600 μmol photons m^−2^ s^−1^. This discrepancy between theoretical and actual LSP is caused by the actual Φ_PSII_, which is related to the consumption rates of ATP and NADPH required for Rubisco carboxylation. As we know, the capacity of Rubisco carboxylation is determined by *V*_cmax_ and chloroplast CO_2_ concentration, the latter of which is tightly related to CO_2_ diffusional conductance (*g*_s_ and *g*_m_) [18,19,20]. Therefore, the light intensity dependence of *A*_N_ is theoretically determined by the light responses of *V*_cmax_, *g*_s_, and/or *g*_m_. In angiosperms, *V*_cmax_ and *g*_s_ usually increase with increasing illumination, but *g*_m_ is maintained stable in some species and gradually increases in others [8,9,21,22,23]. However, it remains unclear whether the light intensity dependence of *A*_N_ is mainly related to *V*_cmax_ or diffusional CO_2_ conductance (*g*_s_ and *g*_m_).

In this study, we measured light response data of gas exchange and chlorophyll fluorescence in twelve tree species with the aim of systematically understanding the main factor influencing the light intensity dependence of *A*_N_. The results indicated that the relative increase in *A*_N_ at saturating light compared with that at sub-saturating light was mainly determined by the increased kinetics of *V*_cmax_ rather than *g*_s_ and *g*_m_. Therefore, optimizing the light intensity dependence of *V*_cmax_ has the potential to enhance crop yield.

## 2. Materials and Methods

### 2.1. Plant Materials and Growth Condition

In this study, we used the seedlings of twelve tree species with variation in the photosynthetic capacity: *Chaenomeles cathayensis* (Hemsl.) C. K. Schneid., *Cercis glabra* Pamp., *Pterocarya stenoptera* C. DC., *Idesia polycarpa* Maxim., *Idesia polycarpa* var. vestita Diels, *Melia azedarach* L., *Pistacia chinensis* Bunge, *Fraxinus retusifoliolata* Feng ex P. Y. Bai, *Hypericum monogynum* L., *Styrax grandiflorus* Griff., *Sunhangia elegans* (DC.) H. Ohashi and K. Ohashi, and *Alnus nepalensis* D. Don. All plants were cultivated in greenhouses in Kunming, Yunnan, China. The day/night temperature is 30/20 °C, relative humidity is about 60%, and the maximum light intensity to which leaves were exposed is approximately 1000 μmol photons m^−2^ s^−1^. To avoid any water and nutrient stress, plants were cultivated using a soilless substrate and drip irrigation techniques.

### 2.2. Gas Exchange and Chlorophyll Fluorescence Measurements

In the morning of clear days in summer, gas exchange and chlorophyll fluorescence were measured at 25 °C using the chlorophyll fluorescence probe of the LI-6400XT portable photosynthesis system (LI-COR Inc., Lincoln, NE, USA). Mature sun leaves were initially exposed to high light (1500 µmol photons m^−2^ s^−1^, composed of 90% red light and 10% blue light) for at least 30 min to complete photosynthetic induction. Subsequently, we measured the light intensity dependence of photosynthesis by gradually decreasing light intensity from 2000 to 100 µmol photons m^−2^ s^−1^. After exposure to each light intensity (2000, 1500, 1000, 600, 400, 200, and 100 µmol photons m^−2^ s^−1^) for 3 min, the data for gas exchange and chlorophyll fluorescence were then recorded.

In our study, we utilized the multi-phase flash (MPF) protocol following standard procedures to determine the parameters of chlorophyll fluorescence [24]. The light intensity for measurement was set at 1 µmol m^−2^ s^−1^, while the maximum flash intensity reached 8000 µmol m^−2^ s^−1^. During the second phase of the MPF, the flash intensity was reduced by 60%, with the three flash phases lasting 0.3 s, 0.7 s, and 0.4 s, respectively. Subsequently, we calculated the effective quantum yield of photochemistry for photosystem II (Φ_PSII_) and the total electron transport rate through photosystem II (ETR) using the following equations [25,26]:

$$\Phi_{PSII}=\frac{\left({F_{m}}^{\prime }-F_{s}\right)}{{F_{m}}^{\prime }}$$

$$ETR=\Phi_{PSII}\times PPFD\times 0.45$$

where PPFD represents the light intensity and 0.45 represents the proportion of light energy absorbed by leaves allocated to photosystem II.

### 2.3. Calculation of the Mesophyll Conductance

Mesophyll conductance (*g*_m_) was calculated by combining the gas exchange data and chlorophyll fluorescence data with the following equation [27]:

$$g_{m}=\frac{A_{N}}{C_{i}-{Г}^{*}(ETR+8\left(A_{N}+R_{d}\right))/(ETR-4\left(A_{N}+R_{d}\right))}$$

where *A*_N_ represents the net photosynthetic rate, *C*_i_ represents the intercellular CO_2_ concentration, Г* represents the CO_2_ compensation point in the absence of mitochondrial respiration, and a typical value of 40 μmol mol^−1^ is used [9]; *R*_d_ represents the dark respiration rate measured at night.

The chloroplast CO_2_ concentration (*C*_c_) was calculated as follows [28]:

$$C_{c}=C_{i}-\frac{A_{N}}{g_{m}}$$

The maximum carboxylation rate of Rubisco (*V*_cmax_) was calculated as follows [18,29]:

$$V_{cmax}=\frac{\left(A_{N}+R_{d})(C_{c}+K_{c}(1+{O/K}_{o})\right)}{\left(C_{c}-{Г}^{*}\right)}$$

where *K*_c_ (404 μmol mol^−1^) and *K*_o_ (278 mmol mol^−1^) are the Rubisco Michaelis–Menten constants for CO_2_ and oxygen, respectively; *O* (210 mmol mol^−1^) is the oxygen concentration in the chloroplasts.

### 2.4. Quantitative Calculation of Photosynthetic Limitation

The quantitative calculation formula for the photosynthetic limiting factor is as follows [15]:

$$L_{s}=\frac{g_{tot}/g_{s}\times \partial A_{N}/\partial C_{c}}{g_{tot}+\partial A_{N}/\partial C_{c}}$$

$$L_{m}=\frac{g_{tot}/g_{m}\times \partial A_{N}/\partial C_{c}}{g_{tot}+\partial A_{N}/\partial C_{c}}$$

$$L_{b}=\frac{g_{tot}}{g_{tot}+\partial A_{N}/\partial C_{c}}$$

where *L*_s_, *L*_m_, and *L*_b_ represent the degree of limitation of photosynthesis by *g*_s_, *g*_m_, and biochemical capacity, respectively, and *g*_tot_ represents the overall CO_2_ diffusive conductance, with *g*_tot_ and ∂*A*_N_/∂*C*_c_ being calculated as follows, respectively:

$$g_{tot}=\frac{g_{s}\times g_{m}}{g_{s}+g_{m}}$$

$${\partial A}_{N}/{\partial C}_{c}=V_{cmax}\frac{{Г}^{*}+K_{c}(1+{O/K}_{o})}{{\left(C_{c}+K_{c}(1+{O/K}_{o})\right)}^{2}}$$

### 2.5. Calculation of Electron Flow for Photorespiration

The rate of electron flow for photorespiration (*J*_O_) was calculated according to the following equation [30]:

$$J_{O}=2/3\times (ETR-4\times (A_{N}+R_{d}))$$

### 2.6. Statistical Analysis

Six independent leaves from six different plants were used for each species. The software SigmaPlot 10.0 was used for graphing and fitting.

## 3. Results

### 3.1. The Steady-State Photosynthesis at Saturating Light

We first measured steady-state photosynthesis at a saturating light intensity of 2000 μmol photons m^−2^ s^−1^. As shown in Table 1, the net CO_2_ assimilation rate (*A*_N_) ranged from 10.9 ± 0.66 to 21.3 ± 0.45 μmol m^−2^ s^−1^, stomatal conductance (*g*_s_) ranged from 0.14 ± 0.01 to 0.62 ± 0.04 mol m^−2^ s^−1^, mesophyll conductance (*g*_m_) ranged from 0.09 ± 0.01 to 0.24 ± 0.04 mol m^−2^ s^−1^, electron transport rate (ETR) ranged from 89.6 ± 2.8 to 175 ± 4.6 μmol m^−2^ s^−1^, and the maximum velocity of Rubisco carboxylation (*V*_cmax_) ranged from 91.5 ± 11 to 199 ± 15 μmol m^−2^ s^−1^. These results indicated that photosynthetic capacity varied substantially among the studied tree species.

### 3.2. Light Intensity Dependence of Photosynthetic Parameters

**Figure 1:**
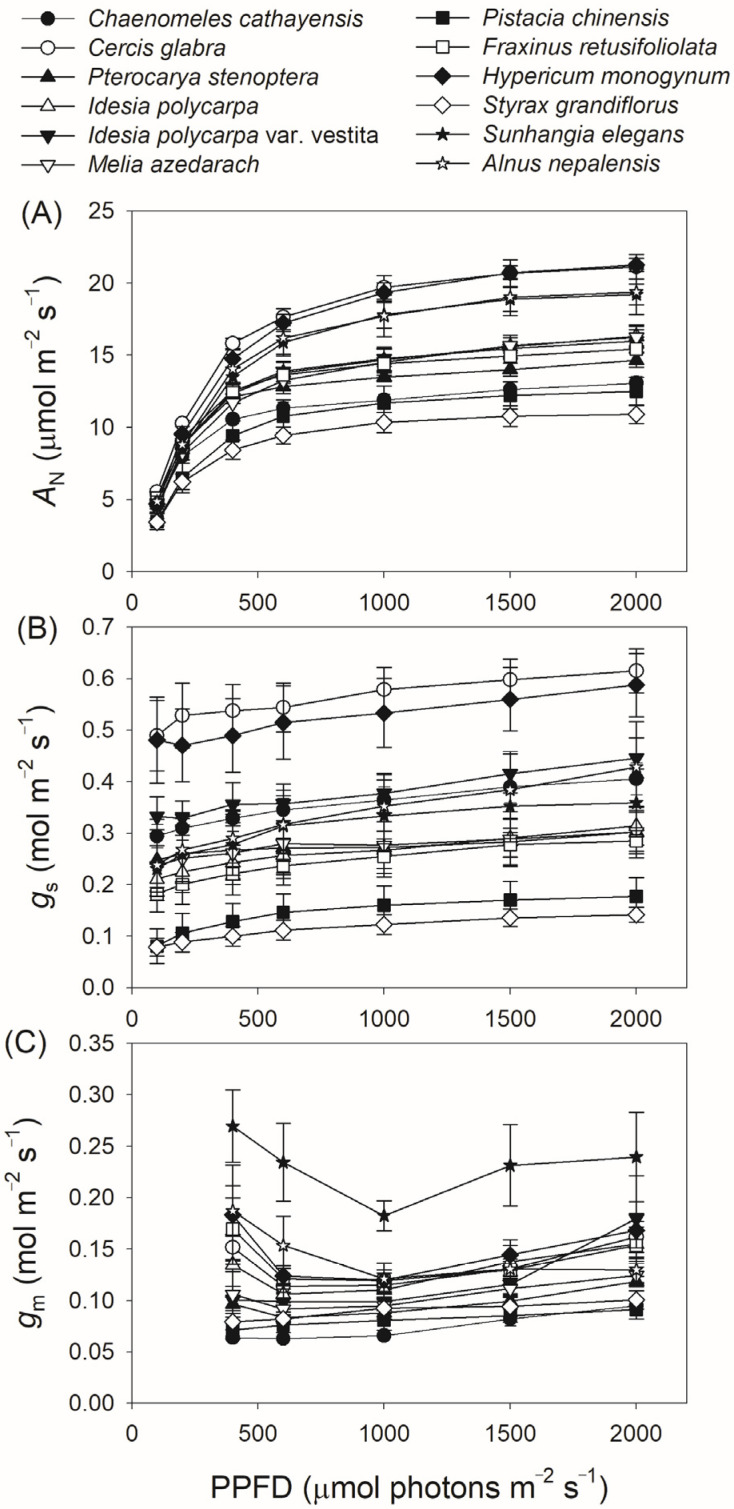
Light intensity dependence of net CO_2_ assimilation rate (*A*_N_) (**A**), stomatal conductance (*g*_s_) (**B**), and mesophyll conductance (*g*_m_) (**C**) in twelve tree species. Data are shown as means ± SE (n = 6).

**Figure 2:**
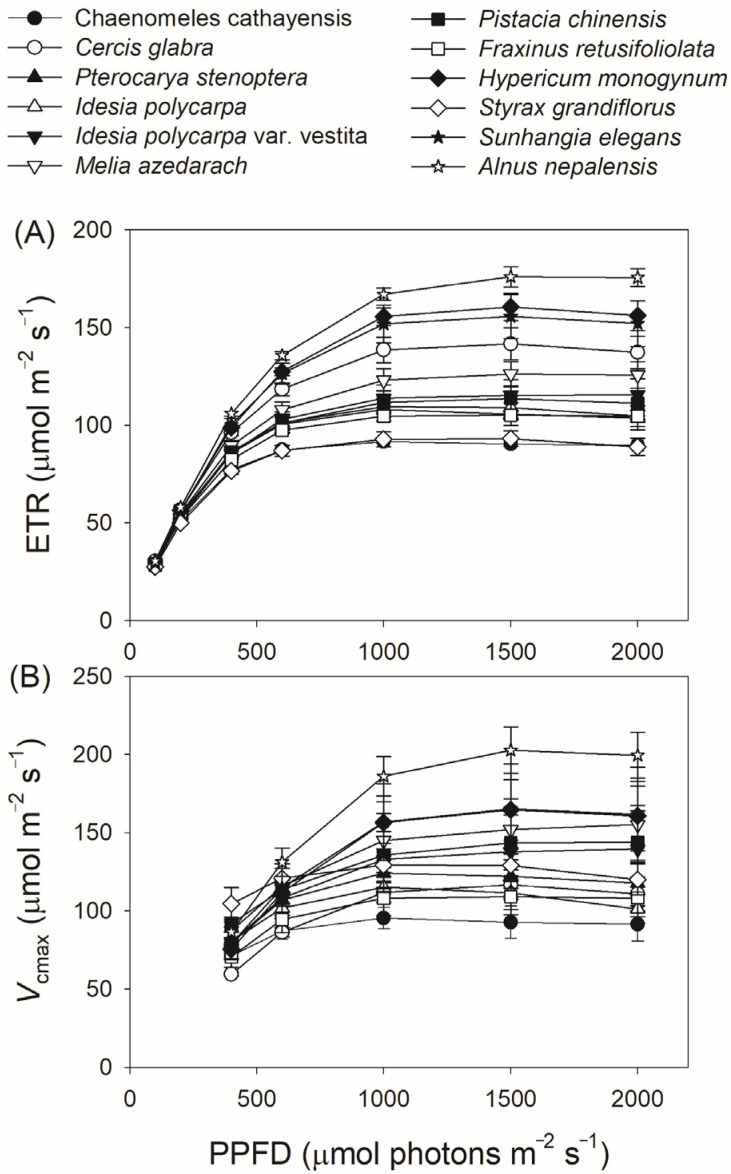
Light intensity dependence of electron transport rate (ETR) (**A**) and the maximum velocity of Rubisco carboxylation (*V*_cmax_) (**B**) in the twelve tree species. Data are shown as means ± SE (n = 6).

**Figure 3:**
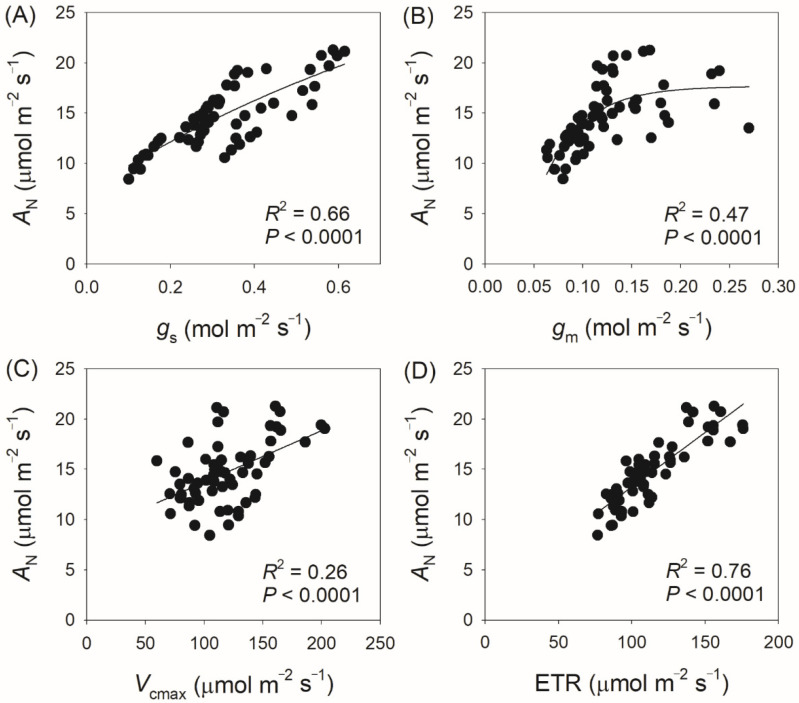
Relationship between *A*_N_, *g*_s_, *g*_m_, *V*_cmax,_ and ETR across light intensities from 400 to 2000 μmol photons m^−2^ s^−1^ among the twelve tree species. *A*_N_, net CO_2_ assimilation rate; *g*_s_, stomatal conductance; *g*_m_, mesophyll conductance; *V*_cmax_, the maximum velocity of Rubisco carboxylation; ETR, electron transport rate. (**A**) relationship between *A*_N_ and *g*_s_; (**B**) relationship between *A*_N_ and *g*_m_; (**C**) relationship between *A*_N_ and *V*_cmax_; (**D**) relationship between *A*_N_ and ETR.

We next measured gas exchange and chlorophyll fluorescence under light conditions ranging from 100 to 2000 μmol photons m^−2^ s^−1^. As shown in Figure 1A, *A*_N_ increased rapidly with increasing light intensity from 100 to 400 μmol photons m^−2^ s^−1^, but increased by an average of 18% when light intensity increased from 600 to 2000 μmol photons m^−2^ s^−1^. At a high light intensity of 1500 μmol photons m^−2^ s^−1^, *A*_N_ was almost saturated in all twelve tree species. Concomitantly, *g*_s_ displayed a slight increase throughout the entire light response curve in all studied species (Figure 1B), while *g*_m_ increased by an average of 32% with increasing light intensity from 600 to 2000 μmol photons m^−2^ s^−1^ (Figure 1C). For all studied species, ETR increased rapidly as light intensity increased from 100 to 600 μmol photons m^−2^ s^−1^. During the light course from 600 to 1000 μmol photons m m^−2^ s^−1^, ETR increased by an average of 12% (Figure 2A). In the twelve studied tree species, ETR was approximately saturated at a high light intensity of 1000 μmol photons m^−2^ s^−1^ (Figure 2A). Similar to the light response trend of ETR, *V*_cmax_ gradually increased with light intensity increasing from 400 to 1000 μmol photons m^−2^ s^−1^, and was almost saturated at 1000 μmol photons m^−2^ s^−1^ in these studied species (Figure 2B). Across the light intensity range from 400 to 2000 μmol photons m^−2^ s^−1^, the variation in *A*_N_ was tightly correlated with the variations in *g*_s_ (*R*^2^ = 0.66, *p* < 0.0001, Figure 3A), *g*_m_ (*R*^2^ = 0.47, *p* < 0.0001, Figure 3B), *V*_cmax_ (*R*^2^ = 0.26, *p* < 0.0001, Figure 3C), and ETR (*R*^2^ = 0.76, *p* < 0.0001, Figure 3D). These results suggest that variation in *A*_N_ among these tree species is related to variations in *g*_s_, *g*_m_, *V*_cmax,_ and ETR.

### 3.3. Correlation Between Physiological Parameters and CO_2_ Assimilation Rate

**Figure 4:**
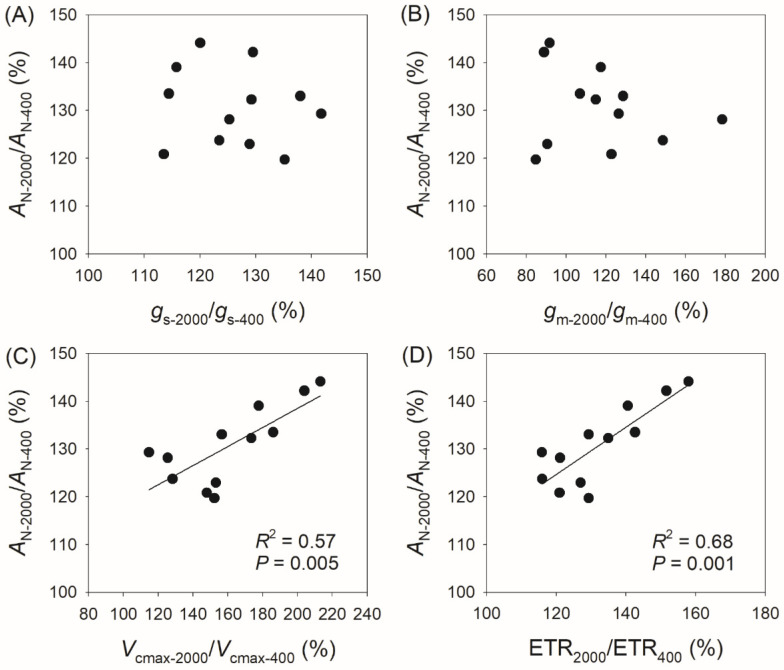
Relationships between *A*_N-2000_/*A*_N-400_, *g*_s-2000_/*g*_s-400_, *g*_m-2000_/*g*_m-400_, *V*_cmax-2000_/*V*_cmax-400,_ and ETR_2000_/ETR_400_ among the twelve tree species. *A*_N-2000_/*A*_N-400_, the ratio of net CO_2_ assimilation rate (*A*_N_) at 2000 μmol photons m^−2^ s^−1^ to that at 400 μmol photons m^−2^ s^−1^; *g*_s-2000_/*g*_s-400_, the ratio of stomatal conductance (*g*_s_) at 2000 μmol photons m^−2^ s^−1^ to that at 400 μmol photons m^−2^ s^−1^; *g*_m-2000_/*g*_m-400_, the ratio of mesophyll conductance (*g*_m_) at 2000 μmol photons m^−2^ s^−1^ to that at 400 μmol photons m^−2^ s^−1^; *V*_cmax-2000_/*V*_cmax-400_, the ratio of maximum velocity of Rubisco carboxylation (*V*_cmax_) at 2000 μmol photons m^−2^ s^−1^ to that at 400 μmol photons m^−2^ s^−1^; ETR_2000_/ETR_400_, the ratio of electron transport rate (ETR) at 2000 μmol photons m^−2^ s^−1^ to that at 400 μmol photons m^−2^ s^−1^. (**A**) relationship between *A*_N-2000_/*A*_N-400_ and *g*_s-2000_/*g*_s-400_; (**B**) relationship between *A*_N-2000_/*A*_N-400_ and *g*_m-2000_/*g*_m-400_; (**C**) relationship between *A*_N-2000_/*A*_N-400_ and *V*_cmax-2000_/*V*_cmax-400_; (**D**) relationship between *A*_N-2000_/*A*_N-400_ and ETR_2000_/ETR_400_.

**Figure 5:**
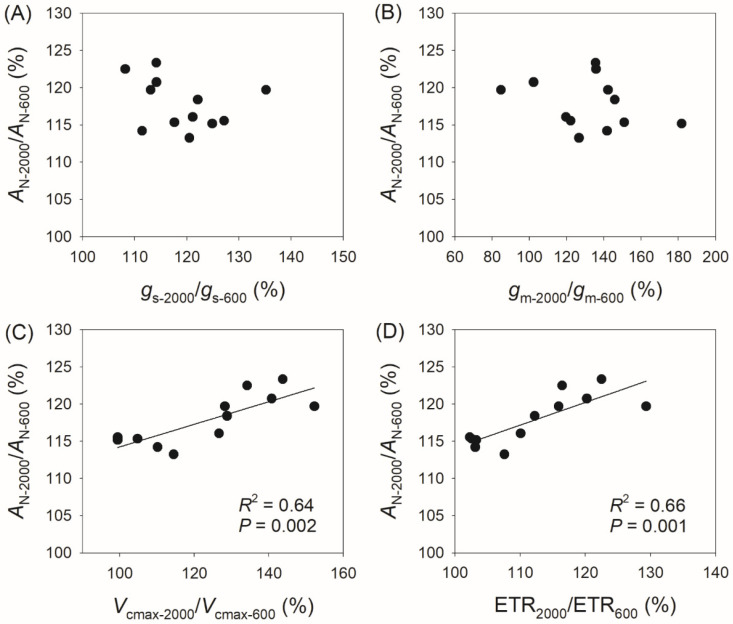
Relationships between *A*_N-2000_/*A*_N-600_, *g*_s-2000_/*g*_s-600_, *g*_m-2000_/*g*_m-600_, *V*_cmax-2000_/*V*_cmax-600_, and ETR_2000_/ETR_600_ among the twelve tree species. *A*_N-2000_/*A*_N-600_, the ratio of net CO_2_ assimilation rate (*A*_N_) at 2000 μmol photons m^−2^ s^−1^ to that at 600 μmol photons m^−2^ s^−1^; *g*_s-2000_/*g*_s-600_, the ratio of stomatal conductance (*g*_s_) at 2000 μmol photons m^−2^ s^−1^ to that at 600 μmol photons m^−2^ s^−1^; *g*_m-2000_/*g*_m-600_, the ratio of mesophyll conductance (*g*_m_) at 2000 μmol photons m^−2^ s^−1^ to that at 600 μmol photons m^−2^ s^−1^; *V*_cmax-2000_/*V*_cmax-600_, the ratio of maximum velocity of Rubisco carboxylation (*V*_cmax_) at 2000 μmol photons m^−2^ s^−1^ to that at 600 μmol photons m^−2^ s^−1^; ETR_2000_/ETR_600_, the ratio of electron transport rate (ETR) at 2000 μmol photons m^−2^ s^−1^ to that at 600 μmol photons m^−2^ s^−1^. (**A**) relationship between *A*_N-2000_/*A*_N-600_ and *g*_s-2000_/*g*_s-600_; (**B**) relationship between *A*_N-2000_/*A*_N-600_ and *g*_m-2000_/*g*_m-600_; (**C**) relationship between *A*_N-2000_/*A*_N-600_ and *V*_cmax-2000_/*V*_cmax-600_; (**D**) relationship between *A*_N-2000_/*A*_N-600_ and ETR_2000_/ETR_600_.

Compared with *A*_N_ at 400 μmol photons m^−2^ s^−1^ (*A*_N-400_), *A*_N_ at 2000 μmol photons m^−2^ s^−1^ (*A*_N-2000_) reached 120–144% in the studied species, with *g*_s_ reaching 114–142%, *g*_m_ reaching 85–178%, *V*_cmax_ reaching 115–213%, and ETR reaching 116–158% (Figure 4). The variation in *A*_N-2000_/*A*_N-400_ was not significantly correlated with *g*_s-2000_/*g*_s-400_ (Figure 4A) or *g*_m-2000_/*g*_m-400_ (Figure 4B), but was significantly correlated with *V*_cmax-2000_/*V*_cmax-400_ (*R*^2^ = 0.57, *p* = 0.005, Figure 4C) and ETR_2000_/ETR_400_ (*R*^2^ = 0.68, *p* = 0.001, Figure 4D). Similarly, the variation in *A*_N-2000_/*A*_N-600_ was not significantly correlated with *g*_s-2000_/*g*_s-600_ (Figure 5A) or *g*_m-2000_/*g*_m-600_ (Figure 5B), but was significantly correlated with *V*_cmax-2000_/*V*_cmax-600_ (*R*^2^ = 0.64, *p* = 0.002, Figure 5C) and ETR_2000_/ETR_600_ (*R*^2^ = 0.66, *p* = 0.001, Figure 5D). Therefore, the relative increase in *A*_N_ at 2000 μmol photons m^−2^ s^−1^ compared with that at 400 and 600 μmol photons m^−2^ s^−1^ is mainly determined by the light intensity dependence of *V*_cmax_ and ETR rather than *g*_s_ and *g*_m_.

**Figure 6:**
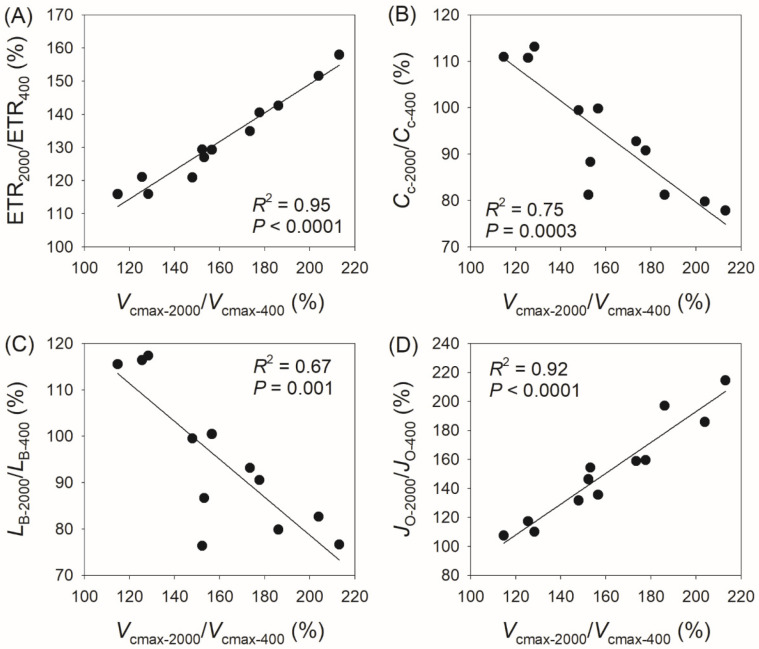
Relationships between *V*_cmax-2000_/*V*_cmax-400_, ETR_2000_/ETR_400_, *C*_c-2000_/*C*_c-400_, *L*_B-2000_/*L*_B-400_, and *J*_O-2000_/*J*_O-400_ among the twelve tree species. *V*_cmax-2000_/*V*_cmax-400_, the ratio of maximum velocity of Rubisco carboxylation (*V*_cmax_) at 2000 μmol photons m^−2^ s^−1^ to that at 400 μmol photons m^−2^ s^−1^; ETR_2000_/ETR_400_, the ratio of electron transport rate (ETR) at 2000 μmol photons m^−2^ s^−1^ to that at 400 μmol photons m^−2^ s^−1^; *C*_c-2000_/*C*_c-400_, the ratio of chloroplast CO_2_ concentration (*C*_c_) at 2000 μmol photons m^−2^ s^−1^ to that at 400 μmol photons m^−2^ s^−1^; *L*_B-2000_/*L*_B-400_, the ratio of biochemical limitation (*L*_B_) at 2000 μmol photons m^−2^ s^−1^ to that at 400 μmol photons m^−2^ s^−1^; *J*_O-2000_/*J*_O-400_, the ratio of photorespiration (*J*_O_) at 2000 μmol photons m^−2^ s^−1^ to that at 400 μmol photons m^−2^ s^−1^. (**A**), relationship between ETR_2000_/ETR_400_ and *V*_cmax-2000_/*V*_cmax-400_; (**B**) relationship between *C*_c-2000_/*C*_c-400_ and *V*_cmax-2000_/*V*_cmax-400_; (**C**) relationship between *L*_B-2000_/*L*_B-400_ and *V*_cmax-2000_/*V*_cmax-400_; (**D**) relationship between *J*_O-2000_/*J*_O-400_ and *V*_cmax-2000_/*V*_cmax-400_.

**Figure 7:**
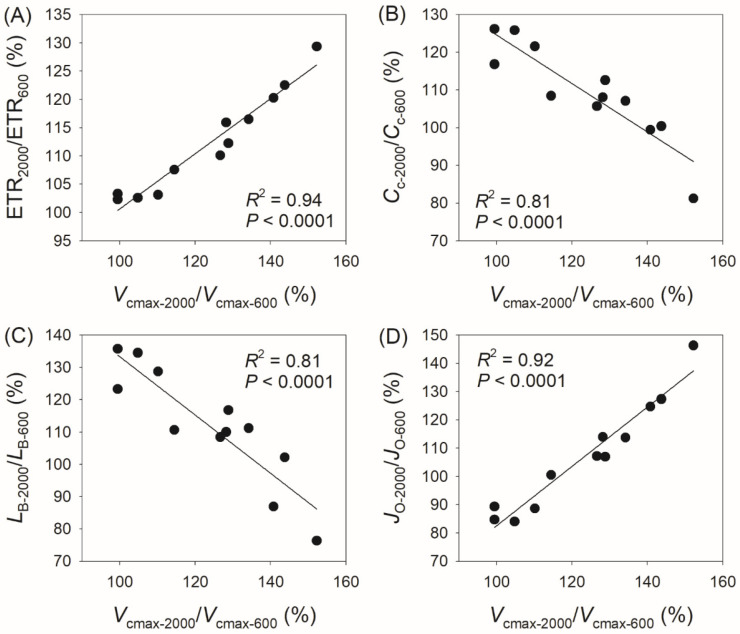
Relationships between *V*_cmax-2000_/*V*_cmax-600_, ETR_2000_/ETR_600_, *C*_c-2000_/*C*_c-600_, *L*_B-2000_/*L*_B-600,_ and *J*_O-2000_/*J*_O-600_ among the twelve tree species. *V*_cmax-2000_/*V*_cmax-600_, the ratio of maximum velocity of Rubisco carboxylation (*V*_cmax_) at 2000 μmol photons m^−2^ s^−1^ to that at 600 μmol photons m^−2^ s^−1^; ETR_2000_/ETR_600_, the ratio of electron transport rate (ETR) at 2000 μmol photons m^−2^ s^−1^ to that at 600 μmol photons m^−2^ s^−1^; *C*_c-2000_/*C*_c-600_, the ratio of chloroplast CO_2_ concentration (*C*_c_) at 2000 μmol photons m^−2^ s^−1^ to that at 600 μmol photons m^−2^ s^−1^; *L*_B-2000_/*L*_B-600_, the ratio of biochemical limitation (*L*_B_) at 2000 μmol photons m^−2^ s^−1^ to that at 600 μmol photons m^−2^ s^−1^; *J*_O-2000_/*J*_O-600_, the ratio of photorespiration (*J*_O_) at 2000 μmol photons m^−2^ s^−1^ to that at 600 μmol photons m^−2^ s^−1^. (**A**) relationship between ETR_2000_/ETR_600_ and *V*_cmax-2000_/*V*_cmax-600_; (**B**) relationship between *C*_c-2000_/*C*_c-600_ and *V*_cmax-2000_/*V*_cmax-600_; (**C**) relationship between *L*_B-2000_/*L*_B-600_ and *V*_cmax-2000_/*V*_cmax-600_; (**D**) relationship between *J*_O-2000_/*J*_O-600_ and *V*_cmax-2000_/*V*_cmax-600_.

In addition, we found a positive correlation between *V*_cmax-2000_/*V*_cmax-400_ and ETR_2000_/ETR_400_ (*R*^2^ = 0.95, *p* < 0.0001, Figure 6A), a negative correlation between *V*_cmax-2000_/*V*_cmax-400_ and *C*_c-2000_/*C*_c-400_ (*R*^2^ = 0.75, *p* = 0.0003, Figure 6B), a negative correlation between *V*_cmax-2000_/*V*_cmax-400_ and *L*_b-2000_/*L*_b-400_ (*R*^2^ = 0.67, *p* = 0.001, Figure 6C), and a positive correlation between *V*_cmax-2000_/*V*_cmax-400_ and *J*_O-2000_/*J*_O-400_ (*R*^2^ = 0.95, *p* < 0.0001, Figure 6D). Similarly, we observed a positive correlation between *V*_cmax-2000_/*V*_cmax-600_ and ETR_2000_/ETR_600_ (*R*^2^ = 0.94, *p* < 0.0001, Figure 7A), a negative correlation between *V*_cmax-2000_/*V*_cmax-600_ and *C*_c-2000_/*C*_c-600_ (*R*^2^ = 0.81, *p* < 0.0001, Figure 7B), a negative correlation between *V*_cmax-2000_/*V*_cmax-600_ and *L*_b-2000_/*L*_b-600_ (*R*^2^ = 0.81, *p* < 0.0001, Figure 7C), and a positive correlation between *V*_cmax-2000_/*V*_cmax-600_ and *J*_O-2000_/*J*_O-600_ (*R*^2^ = 0.92, *p* < 0.0001, Figure 7D). These results indicate that the coordinated changes in *V*_cmax_ and ETR compensate for the decrease in *C*_c_ and the increase in *J*_O_ and diminish the biochemical limitation of *A*_N_.

## 4. Discussion

The light intensity dependence of photosynthesis varies widely among different plants and is tightly related to plant growth and crop yield [11]. According to the C_3_ photosynthesis model, the CO_2_ assimilation rate (*A*_N_) at a given incident light intensity can be limited by light energy (as indicated by ETR), diffusional CO_2_ conductance (*g*_s_ and *g*_m_), and *V*_cmax_ [18]. With increasing illumination from sub-saturating to saturating light, angiosperms typically display gradual increases in ETR and *g*_s_, and *g*_m_ remains stable in some species but gradually increases in others [8,9,21,22]. However, the major factor affecting the light intensity dependence of *A*_N_ remains unclear.

In this study, we found that light intensity dependence of *A*_N_, *V*_cmax,_ and ETR varied among the twelve studied tree species (Figure 1 and Figure 2). Furthermore, the increase in *A*_N_ from sub-saturating to saturating light among the twelve studied tree species was tightly related to changes in *V*_cmax_ and ETR, with no significant correlation to changes in *g*_s_ and *g*_m_ (Figure 4 and Figure 5). Therefore, variation in the light intensity dependence of *A*_N_ among species is primarily determined by biochemical capacity rather than diffusional conductance. Compared with sub-saturating light, the increase in *V*_cmax_ under saturating light suggests an elevation in the Rubisco activation state [31], allowing for increases in Rubisco carboxylation and oxygenation. The ETR-dependent regeneration rates of ATP and NADPH are mainly determined by their consumption rates, which in turn are influenced by primary metabolism, including CO_2_ assimilation and photorespiration [32,33,34]. When CO_2_ assimilation is restricted, the inactivation of chloroplast ATP synthase results in an increase in proton gradient (ΔpH) across the thylakoid membranes, which slows down the electron flow at cytochrome *b*_6_/*f* complex [32,35]. Therefore, the coordination between *V*_cmax_ and ETR tightly determines the light intensity dependence of *A*_N_.

The biological significance of *V*_cmax_ in the light response of *A*_N_ aligns with the general understanding that *V*_cmax_ is an energy-dependent process. For example, *V*_cmax_ gradually increases upon transition from low to high light, imposing a significant on *A*_N_ [22,36]. With increasing maximum *V*_cmax_, the time required to reach 95% of the maximum *V*_cmax_ during light induction also gradually increases [37]. In *Arabidopsis thaliana*, the value of *V*_cmax_ at 1200 μmol photons m^−2^ s^−1^ is 2-fold higher than that at 100 μmol photons m^−2^ s^−1^ [22]. Consistently, we found that *V*_cmax_ at 600 μmol photons m^−2^ s^−1^ was substantially higher than that at 400 μmol photons m^−2^ s^−1^ in the twelve tree species (Figure 2). Furthermore, as *V*_cmax-2000_/*V*_cmax-400_ and *V*_cmax-2000_/*V*_cmax-600_ increased, ETR_2000_/ETR_400_ and ETR_2000_/ETR_600_ also increased simultaneously (Figure 6 and Figure 7), suggesting that ETR provides the essential energy for the increase in *V*_cmax_ through Rubisco activation.

Accompanying the increase in *V*_cmax_ under saturating light, the value of *C*_c_ decreased (Figure 6 and Figure 7), resulting in an inevitable increase in the rate of Rubisco oxygenation (photorespiration). Photorespiration begins with the oxygenation of RuBP catalyzed by Rubisco, producing 2-phosphoglycolate (2-PG). Given that 2-PG inhibits central enzymes in the Calvin–Benson cycle and glycolysis, it must be converted into glycerate-3-P through the photorespiratory pathway. This process requires additional ATP and NADPH produced by ETR. Therefore, *J*_O-2000_/*J*_O-400_ and *J*_O-2000_/*J*_O-600_ positively increased with the increases in *V*_cmax-2000_/*V*_cmax-400_ and *V*_cmax-2000_/*V*_cmax-600_ (Figure 6 and Figure 7). Under conditions of high *V*_cmax_, the rate of RuBP oxygenation was inevitably elevated in normal air. Such enhancement of photorespiration accelerates the consumption rate of ATP and NADPH. This process not only prevents substrate limitation of chloroplast ATP synthase but also favors electron transfer from PSI to NADP^+^ [38,39]. Therefore, photorespiration participates in the light intensity dependence of *V*_cmax_ and ETR.

**Figure 8:**
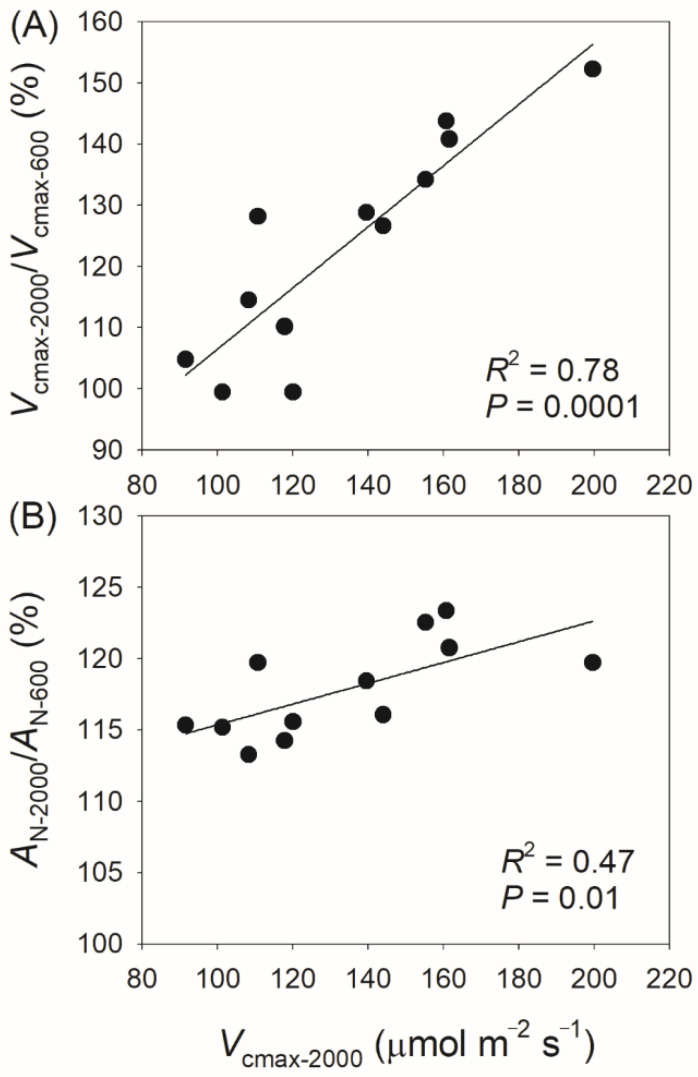
Relationships between *V*_cmax-2000_/*V*_cmax-600_, *A*_N-2000_/*A*_N-600_, and *V*_cmax-2000_ among the twelve tree species. *V*_cmax-2000_/*V*_cmax-600_, the ratio of maximum velocity of Rubisco carboxylation (*V*_cmax_) at 2000 μmol photons m^−2^ s^−1^ to that at 600 μmol photons m^−2^ s^−1^; *A*_N-2000_/*A*_N600_, the ratio of net CO_2_ assimilation rate (*A*_N_) at 2000 μmol photons m^−2^ s^−1^ to that at 600 μmol photons m^−2^ s^−1^; *V*_cmax-2000_, the value of *V*_cmax_ at 2000 μmol photons m^−2^ s^−1^. (**A**) relationship between *V*_cmax-2000_/*V*_cmax-600_ and *V*_cmax-2000_; (**B**) relationship between *A*_N-2000_/*A*_N-600_ and *V*_cmax-2000_.

Considering the strong effect of *V*_cmax_ on the light intensity dependence of *A*_N_, it is surprising that the light intensity dependence of *V*_cmax_ varies among different species (Figure 2). We found that the values of *V*_cmax-2000_/*V*_cmax-600_ and *A*_N-2000_/*A*_N-600_ were positively correlated with the steady-state *V*_cmax_ at 2000 μmol photons m^−2^ s^−1^ (Figure 8). As a result, the light intensity dependence of *V*_cmax_ and *A*_N_ is largely determined by the maximum capacity of *V*_cmax_. In plants with low *V*_cmax_ capacity, such as *Chaenomeles cathayensis*, *V*_cmax_ can reach its maximum value at 600 μmol photons m^−2^ s^−1^ (Figure 2). By comparison, in plants with high *V*_cmax_ capacity, such as *Alnus nepalensis*, *V*_cmax_ saturates at 1500 μmol photons m^−2^ s^−1^ (Figure 2). In wild-type plants, the maximum value of leaf *V*_cmax_ is tightly related to leaf Rubisco content or leaf nitrogen content [5,40,41]. Therefore, variation in the light intensity dependence of *V*_cmax_ and *A*_N_ among tree species may be determined by leaf Rubisco content or leaf nitrogen content.

## 5. Conclusions

We document that the light intensity dependence of *A*_N_ is more related to the light response of *V*_cmax_ rather than the light response of *g*_s_ and *g*_m_. Furthermore, the light response of *V*_cmax_ is in turn influenced by its maximum capacity. Therefore, the maximum *V*_cmax_ plays a significant role in determining the light intensity dependence of *A*_N_. We propose that increasing the maximum *V*_cmax_ or enhancing the nitrogen distribution into Rubisco has the potential to improve *A*_N_ under sub-saturating and saturating light conditions. This strategy provides insight into crop improvement.

## Figures and Tables

**Table 1:** Area-based photosynthetic characteristics for evergreen and deciduous angiosperm tree species. Average values ± SE (n ≥ 5) are shown for area-based maximum net photosynthetic rate (*A*_N_), stomatal conductance (*g*_s_), mesophyll conductance (*g*_m_), maximum velocity of Rubisco carboxylation (*V*_cmax_), and electron transport rate (ETR).

Species	*A*_area_ (μmol m^−2^ s^−1^)	*g*_s_ (mol m^−2^ s^−1^)	*g*_m_ (mol m^−2^ s^−1^)	*V*_cmax_ (μmol m^−2^ s^−1^)	ETR (μmol m^−2^ s^−1^)
*Ch. cathayensis*	13.1 ± 0.43	0.406 ± 0.031	0.095 ± 0.01	91.5 ± 11	89.6 ± 2.8
*Ce. glabra*	21.1 ± 0.85	0.615 ± 0.043	0.16 ± 0.02	111 ± 11	137 ± 8.2
*Pt. stenoptera*	14.7 ± 0.49	0.303 ± 0.042	0.12 ± 0.005	118 ± 14	104 ± 6.0
*Id. polycarpa*	16.3 ± 0.77	0.314 ± 0.037	0.16 ± 0.01	101 ± 9.1	105 ± 5.4
*Id. Polycarpa* var. vestita	16.0 ± 0.78	0.446 ± 0.039	0.18 ± 0.04	139 ± 25	116±8.3
*Me. azedarach*	16.3 ± 0.76	0.302 ± 0.05	0.12 ± 0.01	155 ± 25	126 ± 6.9
*Pi. chinensis*	12.5 ± 0.97	0.177 ± 0.037	0.091 ± 0.01	144 ± 24	111 ± 6.4
*Fr. retusifoliolata*	15.4 ± 0.23	0.285 ± 0.02	0.15 ± 0.02	108 ± 12	105 ± 3.0
*Hy. monogynum*	21.3 ± 0.45	0.588 ± 0.06	0.17 ± 0.02	161± 22	156 ± 7.6
*St. grandiflorus*	10.9 ± 0.66	0.142 ± 0.01	0.10 ± 0.01	120 ± 9.9	88.9 ± 4.3
*Su. elegans*	19.2 ± 0.71	0.359± 0.07	0.24 ± 0.04	162 ± 30	152 ± 12
*Al. nepalensis*	19.4 ± 1.6	0.428 ± 0.09	0.13 ± 0.01	200 ±15	176 ± 4.6

## Data Availability

The data presented in this study are available on request from the corresponding author. Data available on request due to privacy.
